# OsbHLH5 Synergically Regulates Phenolamide and Diterpenoid Phytoalexins Involved in the Defense of Rice Against Pathogens

**DOI:** 10.3390/ijms252212152

**Published:** 2024-11-12

**Authors:** Shen Zhou, Ran Zhang, Qiming Wang, Jinjin Zhu, Junjie Zhou, Yangyang Sun, Shuangqian Shen, Jie Luo

**Affiliations:** 1 National Key Laboratory for Tropical Crop Breeding, School of Breeding and Multiplication (Sanya Institute of Breeding and Multiplication), Hainan University, Sanya 572025, China; zhoushen@yzwlab.cn (S.Z.); zhangran@hainanu.edu.cn (R.Z.); 23210901000025@hainanu.edu.cn (Q.W.); 24110901000032@hainanu.edu.cn (J.Z.); sand_zhou@hainanu.edu.cn (J.Z.); sunyy626@163.com (Y.S.); 2 Yazhouwan National Laboratory, Sanya 572025, China

**Keywords:** phenolamide, diterpenoid, basic helix–loop–helix, metabolic regulation, pathogen resistance

## Abstract

Rice (*Oryza sativa*) produces phenolamides and diterpenoids as major phytoalexins. Although the biosynthetic pathways of phenolamides and diterpenoids in plants have been revealed, knowledge of their accumulation regulatory mechanisms remains limited, and, in particular, no co-regulatory factor has been identified to date. Here, using a combined co-expression and evolutionary analysis, we identified the basic helix–loop–helix (bHLH) transcription factor *OsbHLH5* as a positive bifunctional regulator of phenolamide and diterpenoid biosynthesis in rice. Metabolomic analysis revealed that *OsbHLH5* significantly increased the content of phenolamides (such as feruloyl tryptamine (Fer-Trm) and *p*-coumaroyl tyramine (Cou-Tyr)) and diterpenoid phytoalexins (such as momilactones A, momilactones B) in the overexpression lines, while their content was reduced in the *OsbHLH5* knockout lines. Gene expression and dual-luciferase assays revealed that *OsbHLH5* activates phenolamide biosynthetic genes (including *putrescine hydroxycinnamoyltransferase 3* (*OsPHT3*), *tyramine hydroxycinnamoyltransferases 1/2* (*OsTHT1/2*), and *tryptamine benzoyltransferase 2* (*OsTBT2*)) as well as diterpenoid biosynthetic genes (including *copalyl diphosphate synthase 4* (*OsCPS4*) and *kaurene synthase-like 4/7/10/11* (*OsKSL4/7/10/11*)). Furthermore, we have demonstrated that *OsbHLH5* is induced by jasmonic acid (JA), while pathogen inoculation assays indicated that the overexpression of *OsbHLH5* in transgenic rice plants leads to enhanced resistance to *Xanthomonas oryzae* pv. *oryzae* (*Xoo*). Overall, we have identified a positive regulator of phenolamide and diterpenoid biosynthesis and have demonstrated that biotic stress induces phytoalexin accumulation partly in an *OsbHLH5*-dependent manner, providing new insights into the metabolic interactions involved in pathogen response and offering valuable gene resources for the development, through genetic improvement, of new rice varieties that are resistant to diseases.

## 1. Introduction

Throughout their life cycle, plants produce a plethora of structurally diverse and functionally complex metabolites that play crucial roles in growth, development, and adaptation to environmental changes [1]. Based on structural differences, they can be classified into three major categories: phenylpropanoids, terpenoids, and alkaloids [2]. Phenolamides are a class of nitrogen-containing low-molecular-weight compounds derived from the phenylpropanoid pathway [3]. Phenolamides are a diverse group of plant secondary metabolites formed by the combination of an acyl donor containing a benzene ring and a polyamine group through an amide bond (-CO-NH-). The enzymes that catalyze this step are a class of specific hydroxycinnamoyl transferases, most of which belong to the plant BAHD acyltransferase gene family [4]. Common acyl donors in plants include benzoic acid, cinnamic acid, coumaric acid, caffeic acid, ferulic acid, and sinapic acid; common polyamines include putrescine, cadaverine, agmatine, spermidine, spermine, tyramine, tryptamine, serotonin, and dopamine [5]. Based on the chemical structure of the polyamine group in phenolamides, these compounds can be divided into two major categories: aliphatic phenolamides, such as feruloyl putrescine (Fer-Put) and *p*-coumaroyl putrescine (Cou-Put), and aromatic phenolamides, such as feruloyl tryptamine (Fer-Trm) and *p*-coumaroyl tyramine (Cou-Tyr).

Terpenoids are the largest and most structurally complex class of plant secondary metabolites, composed of isoprene units [6]. Depending on the number of isoprene units in their structure, terpenoids can be classified as monoterpenes, sesquiterpenes, and diterpenes, among others. Common diterpenoid phytoalexins identified in rice include oryzalexin S [7], oryzalexins A-F [8], momilactones A and B [9], phytocassanes A-E [10], and *ent*-10-oxodepressin [11,12,13].

Plant phytoalexins are characterized by their low molecular weight and antimicrobial activity [14]. Phenolamides and diterpenoid phytoalexins, as components that strengthen the plant cell wall and act as antimicrobial compounds, accumulate in response to pathogen induction and play a key role in plant chemical and physical defense responses [15]. Feruloyl tyramine (Fer-Tyr) accumulates in onion roots infected by fungi [16]; chitosan treatment increases the content of *p*-coumaroyl tyramine (Cou-Tyr) in *Solanum lycopersicum* [17]; and feruloyl putrescine (Fer-Put) accumulates in rice leaves infected by rice blast [5]. Studies have shown that phenolamides themselves have inhibitory effects on fungal activity. For instance, Fer-Tyr isolated from garlic roots has a high inhibitory effect on the growth of *Fusarium* mycelium [18], while exogenously added *p*-coumaroyl putrescine (Cou-Put) at different concentrations can inhibit the growth of various fungi, with the inhibitory effect becoming more significant at increasing concentrations [19]. Furthermore, when rice is infected with rice blast disease, diterpenoid phytoalexins accumulate rapidly in resistant rice varieties, effectively inhibiting the spread of the pathogen. Specifically, momilactone A and B strongly suppress the growth of *Magnaporthe oryzae* mycelium [20], and phytocassanes A–E and oryzalexins A–D primarily inhibit the germination of *M. oryzae* spores [21,22]. Additionally, momilactone B, secreted by rice roots into the soil, exhibits allelopathic effects by inhibiting the growth of surrounding plants [23]. *Ent*-10-oxodepressin, which is strongly induced by jasmonic acid (JA), shows good resistance to both rice blast and bacterial blight [13].

In recent years, there has been a series of advancements in the metabolic synthesis of phenolamides in rice. For instance, a comprehensive targeted metabolomics method based on LC-MS was used to identify 28 phenolamides from 529 rice accessions, and an *agmatine hydroxycinnamoyltransferase* (*OsAHT*) and two *putrescine hydroxycinnamoyltransferase* (*OsPHT1/2*) have been cloned from rice [24]. Dong et al. have elucidated the distribution and natural variation of spermidine phenolamides in rice, and identified two regulators of spermidine hydroxycinnamoyltransferase in rice using transgenic materials [25]. Peng studied the metabolic profiles of 11 phenolamide compounds in rice leaves and seeds and cloned two *tryptamine hydroxycinnamoyltransferases* (*OsTHT1/2*) and two *tryptamine benzoyltransferases* (*OsTBT1/2*) from rice [26].

The biosynthetic pathways of diterpenoid phytoalexins in rice have been progressively elucidated. Copalyl diphosphate synthase (OsCPS) and kaurene synthase-like (OsKSL) are important synthases in this process. Recent studies have shown that the *ent*-copalyl diphosphate (*ent*-CDP) synthases OsCPS1 and OsCPS2 have different biological functions [27]. OsCPS1 is involved in gibberellin biosynthesis [28], while OsCPS2 is involved in the biosynthesis of phytoalexins, phytocassanes A–E, and oryzalexins A–F. *OsCPS1* is used for growth and *OsCPS2* is used for defense [29,30]. *OsCPS2* and *OsKSL10* are involved in the synthesis of oryzalexins A–F [31]. *OsCPS2* and *OsKSL7* are involved in the synthesis of phytocassanes A–E [32,33]. *OsCPS4* and *OsKSL8* are involved in the synthesis of oryzalexin S [34]. *OsCPS4* and *OsKSL4* are involved in the synthesis of momilactones A, B [31]. *OsKSL11* has been identified as a stemodene synthase, catalyzing a key step in the biosynthesis of the diterpenoid natural product stemodane family, some of which have antiviral activity [35]. *OsKSL6* encodes an *ent*-isokaurene synthase, responsible for the biosynthesis of oryzadione [36].

Transcription factors can form complex regulatory networks in metabolic pathways, participate in complex biological processes, and play important roles [37]. Based on previous studies, it is known that phenolamide synthesis is regulated by many types of transcription factor families, such as MYB, WRKY, and AP2. These transcription factors participate in the synthesis and regulation of phenolamides both directly and indirectly. *NaMYB8*, strongly induced by JA, regulates the synthesis of aliphatic phenolamides in tobacco by controlling the expression of *putrescine hydroxycinnamyl transferase* (*NaAT*), *spermidine hydroxycinnamyl transferase* (*NaDH29*), and *monospermidine hydroxycinnamyl transferase* (*NaCV86*) [38]. An AP2/ERF family transcription factor, *ORA59*, was successfully isolated by yeast one-hybrid screening using *AtACT* promoter as bait [39]. The biosynthesis of terpenoids is regulated by many types of transcription factor families, such as bZIP, WRKY, and bHLH. Researchers have identified a basic leucine zipper (bZIP) transcription factor, *OsTGAP1*, that is induced by chitooligosaccharides, regulating diterpene synthesis [40]. The transcription factor WRKY45, which is dependent on the salicylic acid signaling pathway, has been reported. Studies have shown that the biosynthetic genes of diterpene phytoalexins (DPs) are regulated in a WRKY45-dependent manner by the synergistic action of salicylic acid and cytokinin [41]. The basic helix–loop–helix (bHLH) transcription factor not only plays important roles in plant growth and secondary metabolism but also participates in a variety of plant stress responses [42,43,44]. Researchers have identified a basic helix–loop–helix (bHLH) transcription factor, *OsbHLH25*, which can regulate the transcriptional levels of DP biosynthetic genes and the accumulation of DPs through overexpression and knockout studies, and this transcription factor can also activate the promoters of DP biosynthetic genes [45]. Phenolamides and diterpenoids, as major phytoalexins in rice, are induced to accumulate by pathogens, but whether their synthesis is coordinately regulated is not clear.

The aim of this study was to investigate the molecular mechanism of the basic helix–loop–helix (bHLH) transcription factor *OsbHLH5* in regulating phenolamide and diterpenoid phytoalexin biosynthesis in rice. By combining metabolomic profiling, biosynthetic gene transcriptional-level analysis, and pathogen inoculation assays, we demonstrate that *OsbHLH5* is transcriptionally induced by JA, functioning as a positive bifunctional regulator of phenolamides and diterpenoids that positively controls the resistance to bacterial blight in rice by activating their key biosynthetic genes. This provides new insights into the metabolic interactions involved in the rice pathogen response.

## 2. Results

### 2.1. Discovery of OsbHLH5 Candidate Target Genes

**Figure 1:**
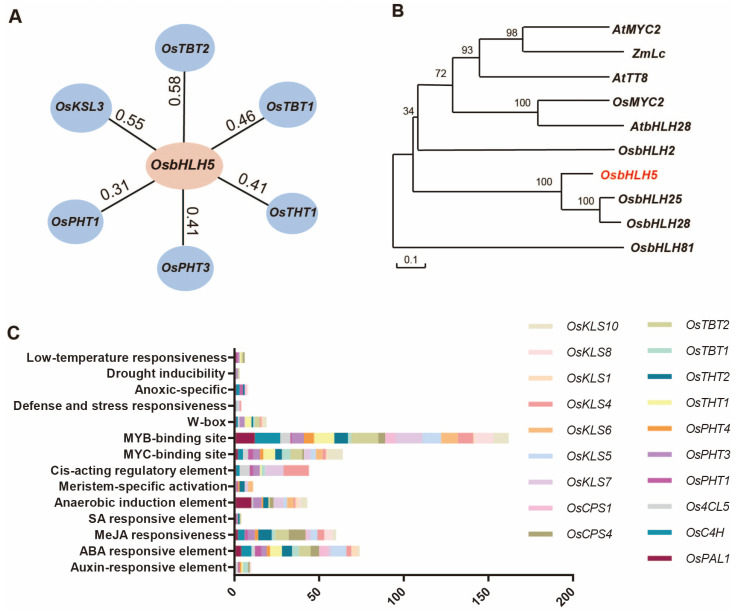
Analysis of candidate target genes regulated by OsbHLH5. (**A**) Networks established from correlations among genes and transcription factors (TFs) of the phenolamide and DP biosynthetic pathways. Pearson correlation coefficient values were calculated for each pair of genes. (**B**) An unrooted phylogenetic tree was constructed as described in the Methods section. The red marking indicates the transcription factor involved in this study–OsbHLH5. Bootstrap values > 70% (based on 1000 replications) are indicated at each node (bar: 0.1 amino acid substitutions per site). (**C**) Promoter analysis of phenolamide and diterpenoid synthesis genes in rice.

The cloning of acyltransferases that regulate phenolamide synthesis in rice marks a significant advancement in elucidating the metabolic pathway involved in phenolamide production (Figure S2) [26]. However, the regulatory mechanism of phenolamides in rice remains an area of ongoing investigation. The discovery of regulatory transcription factors may facilitate the efficient regulation of plant metabolic pathways through the manipulation of individual genes within the plant genome. To identify potential transcription factors that regulate phenolamide synthesis, we examined the promoters of genes involved in this pathway in rice. Our analysis revealed that the promoters of upstream phenolamide synthesis genes, such as *OsPAL1*, *OsC4H*, and *Os4CL5*, as well as those of BAHD acyltransferase genes, including *OsPHT1/3/4*, *OsTHT1/2*, and *OsTBT1/2*, are adorned with a variety of cis-regulatory elements. Among these, the most abundant are transcription-factor-binding sites (including MYB, MYC, and W-box elements), followed by hormone response elements (such as MeJA, ABA, and SA), and a small number of abiotic stress response elements (such as those involved in responses to drought, low temperature, and hypoxia) (Figure 1C) (Table S4). This indicates that the synthesis of phenolamides in rice is subject to intricate regulatory mechanisms.

It is noteworthy that the co-expression analysis of these genes using the RiceFREND database (http://ricefrend.dna.affrc.go.jp/ (accessed on 8 November 2024)) showed that the rice transcription factor bHLH family member *LOC_Os01g09900* is co-expressed with at least five of the acyltransferase genes. According to the GRASSIUS database (https://grassius.org/ (accessed on 8 November 2024)), it is designated as *OsbHLH5* [46]. The co-expression coefficient of *OsbHLH5* with *OsTBT2* was 0.58, and with *OsPHT1* it was 0.31 (Figure 1A) (Table S1). Given that *OsbHLH5* was co-expressed with these BAHD acyltransferases and that the acyltransferase promoter is rich in bHLH binding sites (MYC elements), it can be inferred that *OsbHLH5* may be involved in the transcriptional regulation of rice BAHD acyltransferases, thereby influencing the synthesis of phenolamides.

Phylogenetic trees can reflect the evolutionary relationships between genes, and the analysis of these trees can predict the functions of unknown genes based on the known functions of others. We collected homologs of *OsbHLH5* from various species, including *Oryza sativa*, *Arabidopsis thaliana*, and *Zea mays*, in the NCBI database. The phylogenetic analysis indicated that *OsbHLH5* is in the same evolutionary branch as *OsbHLH25* and *OsbHLH28* in rice, with *OsbHLH25* already reported to positively regulate the biosynthesis of diterpenoid phytoalexins such as oryzalexins and phytocassanes (Figure 1B) [45]. Furthermore, promoter and co-expression analyses of diterpenoid phytoalexin biosynthetic genes revealed that the co-expression coefficient between *OsKSL3* and *OsbHLH5* is 0.55 (Figure 1A). Additionally, the promoters of the diterpenoid phytoalexin synthesis genes *OsCPS* and *OsKSLs* contain a multitude of bHLH recognition elements (Figure 1C). These results suggest that *OsbHLH5* may possess potential dual functionality, participating in the regulation of the synthesis of both phenolamides and diterpenoid phytoalexins.

### 2.2. Subcellular Localization and Expression Pattern Analysis of OsbHLH5

**Figure 2:**
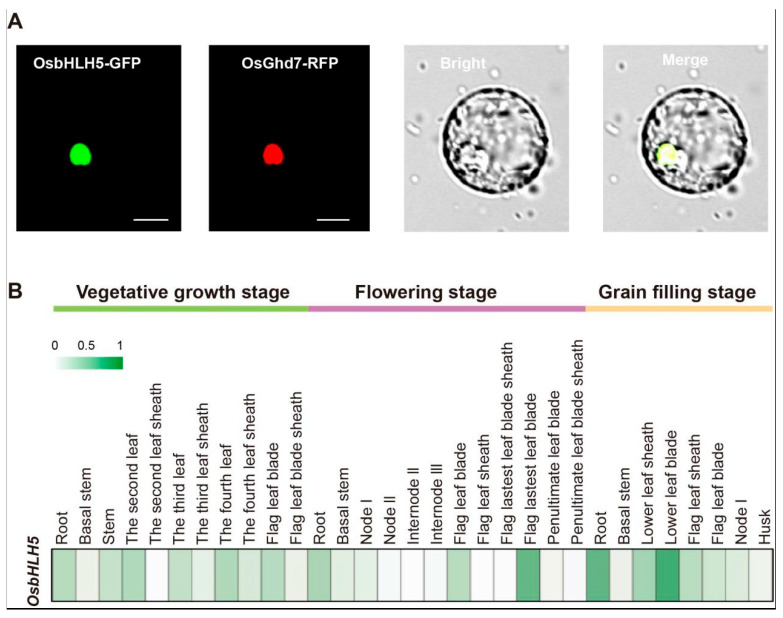
Subcellular localization and expression pattern analysis of *OsbHLH5*. (**A**) Subcellular localization pattern of OsbHLH5. Transient expression of OsbHLH5 fused to green fluorescent protein (GFP) in rice leaf protoplasts. OsGhd7-RFP as a nuclear marker (scale bar, 10 µm), repeated three times. (**B**) Expression pattern analysis of *OsbHLH5*. All tissues were sampled from a widely used rice variety, Zhonghua11, grown in a paddy field. The expression level was determined using RT–qPCR. Data are means ± SD (*n* = 3).

Transcription factors usually act as nuclear proteins and play a role in regulating the transcription of downstream genes in the nucleus. In order to determine the subcellular localization of OsbHLH5, we first used the WoLF PSORT (https://wolfpsort.hgc.jp/ (accessed on 8 November 2024)) online database for the analysis, which showed that OsbHLH5 is mainly engaged in the cell nucleus. To further confirm this result, we performed subcellular localization experiments using protoplast materials from twelve-day-old rice seedlings. An *OsbHLH5*: *GFP* vector was constructed by connecting the N-terminal of the full-length OsbHLH5 sequence (without the stop codon) with GFP, and the rice protoplasts were transformed together with the nuclear marker vector *Ghd7*::*RFP*. Consistent with the expected results, the green fluorescence was co-localized with the red fluorescence, thus confirming OsbHLH5 as a nuclear protein (Figure 2A).

The function of *OsbHLH5* is closely related to its expression tissues and periods. In order to determine the expression pattern of *OsbHLH5* in rice, we selected samples from different tissues of ZH11 in rice at different periods, including roots, stems, leaves, leaf sheaths, and nodes, at the vegetating, flowering, and filling stages of rice. Through RNA extraction and reverse transcription, the obtained cDNA was used as the template, and rice *OsUbc13* was used as the internal control gene to detect the expression of *OsbHLH5* in different parts of the rice. The results show that the expression level of *OsbHLH5* was higher in the leaf and leaf sheath, followed by the root, and was lowest in the node (Figure 2B). In addition, the expression level of *OsbHLH5* was higher during the vegetative growth stage and filling stage than in the flowering stage.

### 2.3. Metabolic Regulatory Network Analysis of OsbHLH5

**Figure 3:**
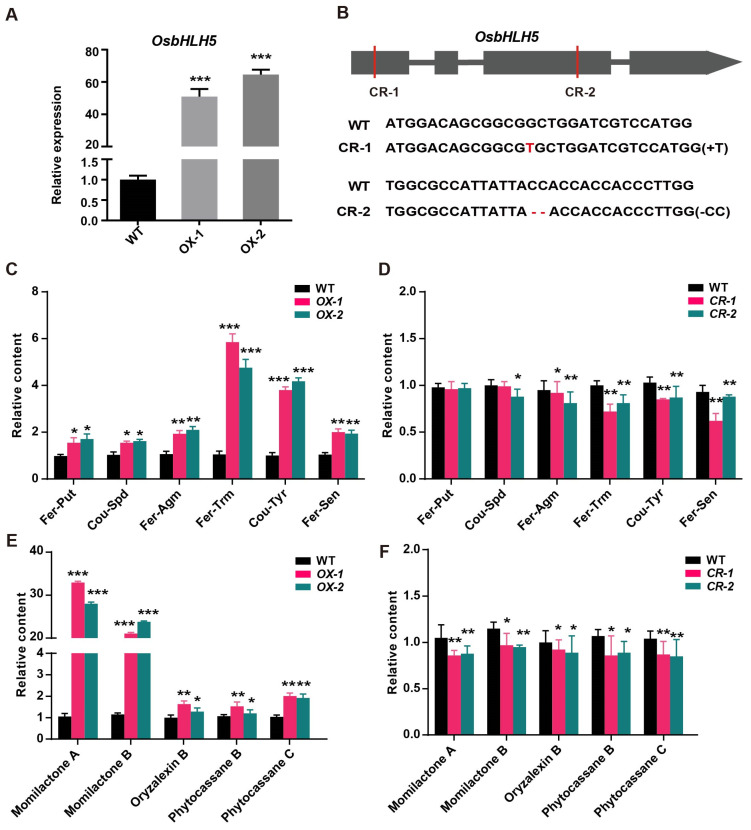
Metabolite analysis of *OsbHLH5* transgenic individuals in vivo. (**A**) Expression analysis of *OsbHLH5* in the overexpression (ox) of transgenic lines; the rice *OsUbc13* gene was used as the internal control. The data are presented as mean ± SD, *n* = 3. (**B**) CRISPR/Cas9 target PAM sequence and edited types for *OsbHLH5*. CR, CRISPR. (**C**,**D**) Bar plots for the content of phenolamides in the OX (**C**) and mutant lines (**D**) of *OsbHLH5*. Fer-Put, feruloyl-putrescine; Cou-Spd, *p*-coumaroyl-spermidine; Fer-Agm, feruloyl-agmatine; Fer-Trm, feruloyl-tryptamine; Cou-Tyr, *p*-coumaroyl-tyramine; Fer-Sen, feruloyl-serotonin. The data are presented as mean ± SD, *n* = 3. (**E**,**F**) Bar plots for the content of diterpenoids in the OX (**E**) and mutant lines (**F**) of *OsbHLH5*. The data are presented as mean ± SD, *n* = 3, * *p* < 0.05, ** *p* < 0.01, *** *p* < 0.001, Student’s *t* tests.

To validate the function of *OsbHLH5* in rice, we constructed an overexpression vector of *OsbHLH5* driven by the maize Ubiquitin promoter. After correct sequencing, a rice genetic transformation operation was carried out through callus infection mediated by *Agrobacterium* EHA105 [47]. Transgenic plants of T2 generation were identified via PCR. qRT-PCR was used to detect gene expression, and the results show that the transcription expression level of *OsbHLH5*-OX (overexpression) lines was significantly higher than that of the wild type (Figure 3A). At the same time, we used CRISPR-Cas9 technology to construct *OsbHLH5* knockout material [48]. As shown, we successfully obtained two *OsbHLH5* mutant materials with different mutation types. *Osbhlh5*-CR1 (CR, CRISPR) has a single T base insertion on the first exon of the gene. *Osbhlh5*-CR2 is a deletion of CC bases on the second exon of the gene, both of which cause frameshift mutations in the gene (Figure 3B).

To test whether *OsbHLH5* is involved in the regulation of the accumulation of phenolamides and diterpenoids in rice, we examined the accumulation pattern of metabolites in both *OsbHLH5*-OX and *Osbhlh5*-CR mutant lines. The results reveal that, compared with the wild type, there were different degrees of accumulation of various phenolamides in the *OsbHLH5*-OX lines. More specifically, the levels of aromatic phenolamides, including feruloyl-tryptamine (Fer-Trm), *p*-coumaroyl-tyramine (Cou-Tyr), and feruloyl-serotonin (Fer-Sen), showed a noticeable upward trend, while the changes in aliphatic phenolamides, such as feruloyl-putrescine (Fer-Put), *p*-coumaroyl-spermidine (Cou-Spd), and feruloyl-agmatine (Fer-Agm), were slightly weaker (Figure 3C). Additionally, we confirmed this trend in the mutant material’s *Osbhlh5*-CR lines, where the accumulation of corresponding phenolamides was generally reduced (Figure 3D). These results indicate that *OsbHLH5* is involved in the regulation of the accumulation of phenolamides in rice and may primarily affect the levels of aromatic phenolamides.

To assess whether *OsbHLH5* is involved in the regulation of diterpenoid phytoalexin accumulation in rice, metabolic profiling analysis was conducted and the results were found to reveal that the *OsbHLH5*-OX lines exhibited significantly higher accumulation levels of diterpenoid phytoalexins (Momilactone A, Momilactone B, Oryzalexin B, Phytocassane B, Phytocassane C) in rice leaves compared with wild-type plants (Figure 3E). Notably, the levels of Momilactone A/B in *OsbHLH5*-OX plants were more than 20-fold higher than those in wild-type leaves. Additionally, we observed a significant downregulation of some diterpenoid metabolites in the *Osbhlh5*-CR mutants (Figure 3F), indicating that *OsbHLH5* can modulate the biosynthesis of diterpenoid secondary metabolites in rice. Collectively, these results suggest that *OsbHLH5* may have a bifunction, participating in the regulation of the accumulation of both phenolamides (particularly aromatic phenolamides) and diterpenoid phytoalexins in rice.

### 2.4. Analysis of Regulatory Mechanisms of OsbHLH5

**Figure 4:**
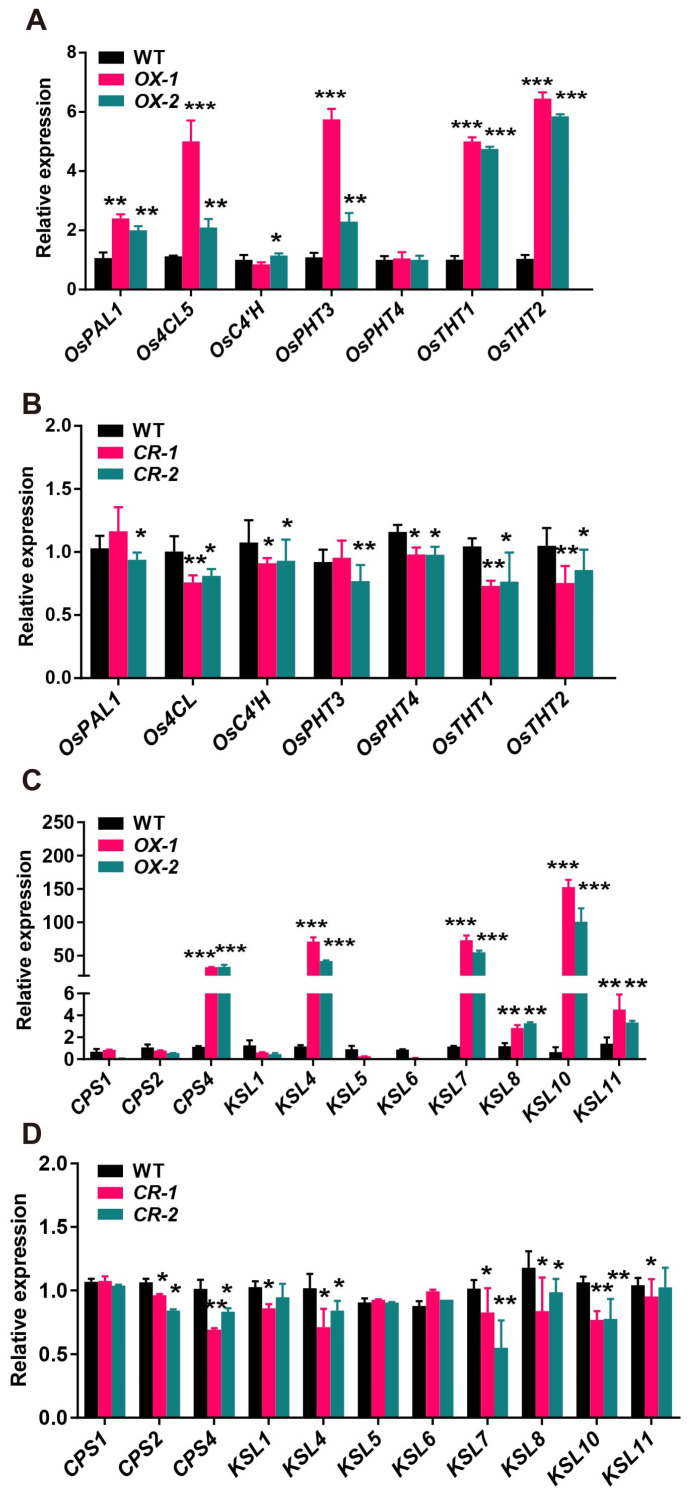
Expression pattern analysis of genes involved in phenolamide and diterpenoid biosynthetic pathway in the overexpression (OX) and mutant lines (CR) of *OsbHLH5*. (**A**,**B**) Expression analysis of phenolamide biosynthetic genes in the OX (**A**) and mutant lines (**B**) of OsbHLH5. The data are presented as mean ± SD, *n* = 3. (**C**,**D**) Expression analysis of diterpenoid biosynthetic genes in the OX (**C**) and mutant lines (**D**) of OsbHLH5. The data are presented as mean ± SD, *n* = 3. The rice OsUbc13 gene was used as the internal control. Asterisks indicate values that are significantly different from the control; * *p* < 0.05, ** *p* < 0.01, *** *p* < 0.001, Student’s *t* test.

*OsbHLH5* regulates the accumulation of phenolamides and diterpenoid phytoalexins in rice. To investigate the underlying molecular mechanisms, we initially examined the expression changes of phenolamide biosynthetic genes in the transgenic materials. Using ZH11 as the wild-type control, we selected two independent transgenic families and collected mature leaf samples at the fifth-leaf stage. The total RNA was extracted and reverse-transcribed. As shown in Figure 4, in the *OsbHLH5*-OX, the expression levels of genes encoding acyltransferases such as *OsTHT1*, *OsTHT2*, and *OsTBT2*, which control the synthesis of aromatic phenolamides, increased by 5–6-fold relative to the wild type (WT), while the expression levels of genes controlling the synthesis of aliphatic phenolamides, including *OsPHT4*, showed no significant change, and *OsPHT3* expression levels increased by approximately three times on average (Figure 4A). In the *Osbhlh5*-CR1/2 materials, the transcription levels of phenolamide biosynthetic genes were significantly decreased (Figure 4B). In summary, in the *OsbHLH5*-OX, the expression levels of certain acyltransferases and key enzymes in the upstream phenylpropanoid pathway increased, while in the *Osbhlh5*-CR, gene expression levels decreased, which corresponds with the accumulation results of phenolamides, proving that *OsbHLH5* may regulate the accumulation of phenolamides in rice by modulating acyltransferases or key enzymes in the upstream phenylpropanoid biosynthetic pathway. Similarly, we detected the expression levels of diterpenoid phytoalexin biosynthetic genes, and the results show that the expression levels of *OsCPS4* and *OsKSL4*, which are mainly responsible for the biosynthesis of momilactones in rice, were significantly increased in the *OsbHLH5*-OX and significantly decreased in the *Osbhlh5*-CR. Concurrently, the expression levels of OsKSL7/8/10/11 also increased to varying degrees in the *OsbHLH5*-OX (Figure 4C,D and Figure S3).

**Figure 5:**
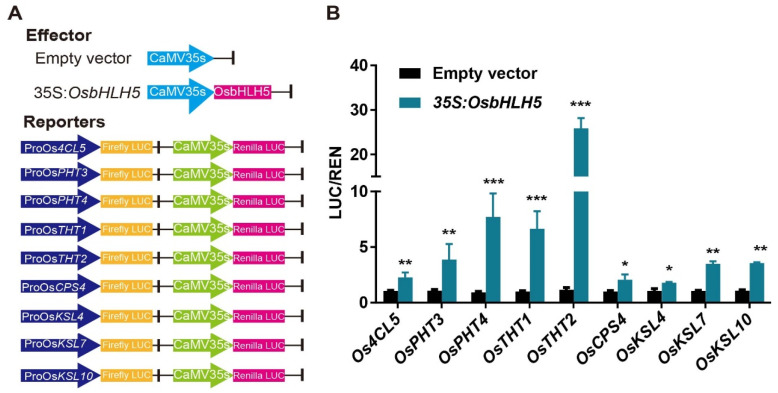
OsbHLH5 positively regulates genes involved in phenolamides and diterpenoid biosynthesis in rice. (**A**) Schematic diagram of the effector and reporter plasmids used in the transient assay in leaf epidermal cells of *N. benthamiana*. Ren, Renilla luciferase; LUC, firefly luciferase. (**B**) OsbHLH5 activates the transcription of biosynthetic genes for phenolamides (*Os4CL5*, *OsPHT*s, and *OsTHT*s) and diterpenoids (*OsCPS4*, *OsKCL4*, *OsKCL7*, and *OsKCL10*). *N. benthamiana* leaves were infiltrated with different combinations of effectors and reporters. The LUC activity was normalized to the REN activity as an internal control. The *p*-value was calculated using Student’s *t* test, *n* = 3. * *p* < 0.05, ** *p* < 0.01, *** *p* < 0.001.

**Figure 6:**
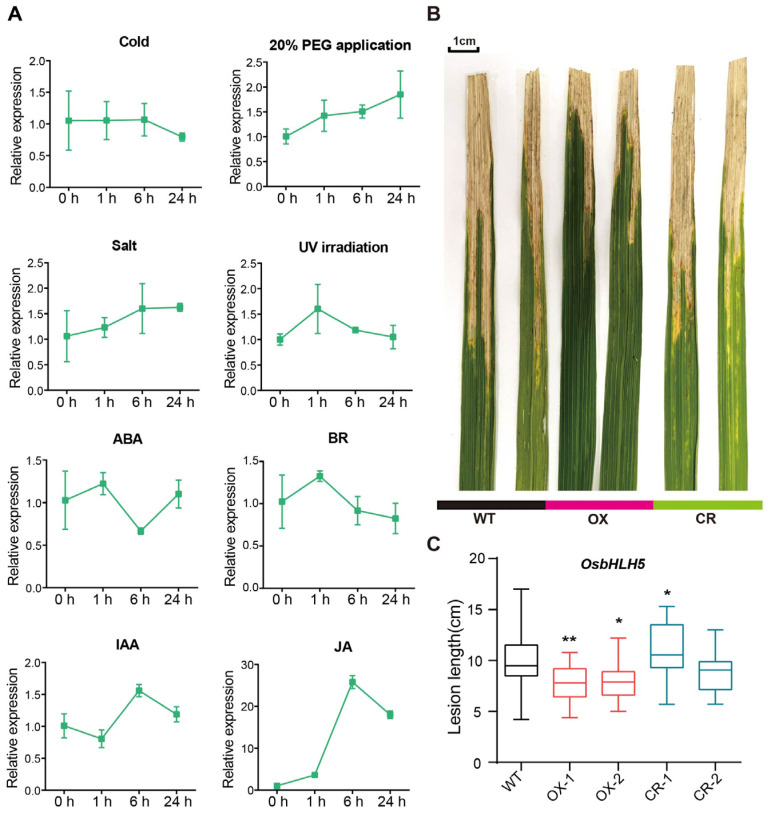
Functional validations of *OsbHLH5* in plants. (**A**) Gene expression profiling of *OsbHLH5* under low temperature (4 °C), 20% PEG, 200 mM NaCl, UV-B, 100 µM ABA, 10 µM BR, 100 µM IAA, and 100 µM JA treatment. The rice *OsUbc13* gene was used as the internal control. h, hour. The data are presented as mean ± SD, *n* = 3. (**B**,**C**) Function of *OsbHLH5* transgenic individuals on *Xoo* pathogen interaction. Typical leaves were photographed (**B**) and the lesion length (**C**) was counted after infection with the *Xoo*-PXO99 strains for 14 days. Ten replicates for lesion length. Bars represent mean ± SD, *n* = 3; * *p* < 0.05, ** *p* < 0.01, Student’s *t* test.

We utilized the dual-luciferase system in tobacco to verify the transcriptional activation activity of *OsbHLH5*. The full-length cDNA sequence of *OsbHLH5* was constructed into the pEAQ-HT vector as the effector plasmid, with the empty vector serving as a control. The reporter plasmid was pH2GW7_REN_LUC, driven by the TATA box and containing the firefly LUC reporter gene, with fragments of the BAHD gene promoters as well as the promoters of *OsCPS4*, *OsKSL4*, and *OsKSL7* inserted in front of the TATA box. Additionally, the reporter plasmid carried the Renilla luciferase gene driven by the 35S promoter as an internal reference (Figure 5A). After transforming the constructed vectors into Agrobacterium EHA105, they were injected into tobacco leaves. The results show that, compared with the empty vector (Control, CK), the OsbHLH5 protein exhibited transcriptional activation activity towards all reporter gene promoters (Figure 5B). OsbHLH5 had a strong activating effect on the BAHD gene promoters involved in phenolamide synthesis genes, with the strongest activity on the promoter of the key gene *OsTHT2* for aromatic phenolamide synthesis, which reached approximately 30 times that of the control and which corresponds with the result obtained when *OsbHLH5* was used to promote the accumulation of Fer-Trm in rice. Furthermore, OsbHLH5 also showed an activation effect of about 1.5–3 times that of the diterpenoid phytoalexin biosynthetic genes *OsCPS4*, *OsKSL4*, and *OsKSL7* (Figure 6B). These results indicate that *OsbHLH5* can positively regulate the transcriptional levels of phenolamides and diterpenoid phytoalexin biosynthetic gene promoters.

### 2.5. OsbHLH5 Positively Regulates Pathogen Resistance in Rice

In order to further reveal the biological processes involved in *OsbHLH5* in rice, the induced expression of *OsbHLH5* under abiotic stress, such as low temperature (4 °C), 20% PEG, 200 mM NaCl, and UV-B, and with hormones, such as ABA, BR, IAA, and JA, was assessed. We took approximately two-week-old ZH11 seedlings and treated them. Then, the samples were taken after 0, 1, 6, and 24 h, to detect the changes in *OsbHLH5* expression. The results show that *OsbHLH5* was not affected by abiotic stress, and was not sensitive to ABA, BR, or IAA. However, it was strongly induced by JA, and the expression level increased by more than 20 times after JA treatment for 6 h (Figure 6A). JA is an important plant hormone that mediates plant responses to herbivores and necrotic pathogen attacks [49], and *OsbHLH5* is induced by JA. Combined with the findings from previous studies in our laboratory, it was found that the acyltransferase and terpenoid synthesis genes controlling phenolamide synthesis in rice are also induced by JA [50,51,52]. These results suggest that JA may be involved in the regulation of acyltransferase and terpenoid synthesis genes in rice via their inducing of *OsbHLH5*.

**Figure 7:**
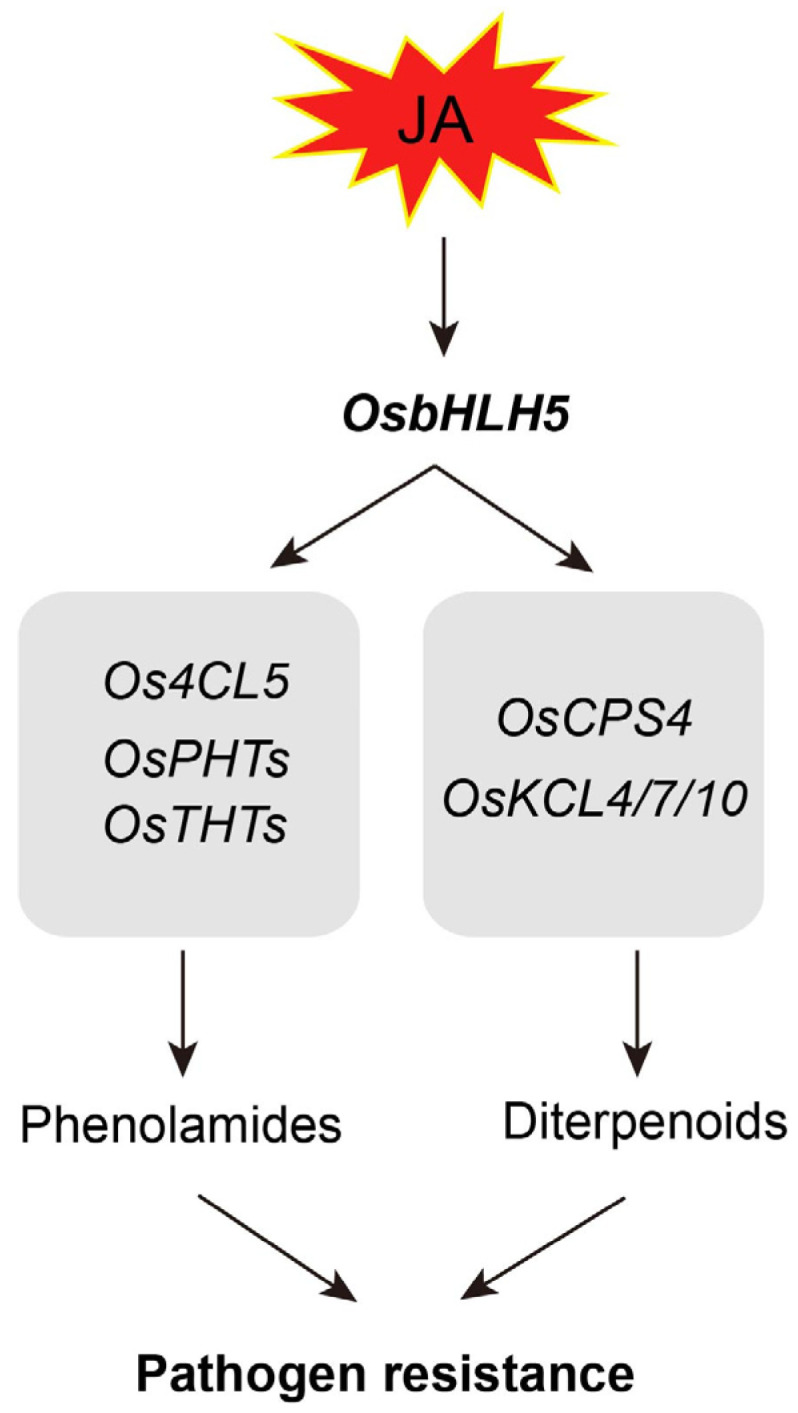
A proposed working model of the role of OsbHLH5, which synergistically activates the biosynthesis of phenolamides and diterpenoids, conferring pathogen resistance in rice.

To verify the potential role of *OsbHLH5* in resistance to pathogens, we inoculated the *OsbHLH5*-OX and *Osbhlh5*-CR lines with the bacterial pathogen *Xanthomonas oryzae* pv. *oryzae* (*Xoo*) PXO99, and all *OsbHLH5*-OX lines were found to have developed smaller lesions after 14 days of inoculation, with a reduced lesion length of approximately 50% compared with the wild type (Figure 6B,C). However, the *Xoo* lesions of the *Osbhlh5*-CR lines were broadly similar to those of the wild type. In conclusion, OsbHLH5 confers positive resistance against Xoo in rice, and the overexpression of *OsbHLH5* enhances resistance to *Xoo*. Taken together, these results indicate that the plant phenylpropanoid metabolite (especially aromatic phenolamides) and diterpenoid levels determined by *OsbHLH5* play an important role in pathogen resistance (Figure 7).

## 3. Discussion

Gene expression regulation is generally carried out by transcription factors, and the elucidation of plant regulatory networks will aid in the future utilization of plant genetic resources. Phenolamides are a class of important plant secondary metabolites closely associated with plant growth, development, and responses to various biotic and abiotic stresses [53,54]. The biosynthetic pathways of phenolamides have been extensively studied in different plants, but research on their regulatory mechanisms is still limited [55,56,57]. Based on previous studies, it is known that the synthesis of phenolamides in plants is regulated by many families of transcription factors, such as MYB, WRKY, AP2, and APIP5 (bZIP) [38,58,59]. These transcription factors are involved in the synthesis and regulation of phenolamides either directly or indirectly. For instance, transcription factors such as NaMYB8, ORA59, HvMYB8, and APIP5 (bZIP) can activate or repress the direct synthesis genes of phenolamides such as the BAHD family acyltransferases [38], whereas StWRKY1 can regulate the upstream genes of phenolamide synthesis, such as *St4CL*, to indirectly promote the synthesis of phenolamides [60]. In recent years, our laboratory has successively cloned several BAHD family acyltransferase genes that control the synthesis of phenolamides in rice [24,25,26]. The co-expression analysis of acyltransferase genes involved in phenolamide synthesis revealed that *OsbHLH5* can be co-expressed with different acyltransferase genes (Figure 1A). To verify whether *OsbHLH5* has the ability to regulate the synthesis of phenolamides, we constructed *OsbHLH5* overexpression materials and CRISPR mutant materials in rice. The detection of phenolamides and related genes showed that both the transcription and metabolic levels of phenolamide-related genes and metabolites increased accordingly in rice, preliminarily confirming that *OsbHLH5* can participate in the regulation of plant phenolamide synthesis. Additionally, in the CRISPR materials of *OsbHLH5*, the content of phenolamides and the expression level of phenolamide synthetic genes decreased. To explain the mechanism of action of *OsbHLH5* at the molecular level, we used the dual-luciferase assay to demonstrate that *OsbHLH5* can activate the promoters of genes such as *OsTHT2* and *OsPHT3*, positively regulating their expression. In this study, we cloned a new bHLH transcription factor that participates in the regulation of phenolamide synthesis in rice for the first time. *OsbHLH5* not only regulates the upstream gene of phenolamide synthesis, *Os4CL5*, but also controls the direct synthesis genes of phenolamides, such as *OsTHT2* and *OsPHT3*, to comprehensively promote the accumulation of phenolamides.

Phylogenetic tree analysis indicates that *OsbHLH5* and *OsbHLH25* are located in the same evolutionary sub-branch, and protein sequence analysis shows that both OsbHLH5 and OsbHLH25 proteins are classified as G box binding factors because they have a typical triad of residues, His, Glu, and Arg, in their basic regions (Figure S1) [61]. Studies have shown that *OsbHLH25* plays an important role in the biosynthesis of diterpenoid phytoalexins in rice [45]. *OsbHLH25* is co-expressed with the synthesis genes of diterpenoid phytoalexins such as *KSL7*, *CPS4*, and *KSL4*, and the overexpression of *OsbHLH25* leads to a significant accumulation of phytocassanes and momilactones. Furthermore, *OsbHLH25* is transcriptionally induced under various stresses, including CuCl_2_, UV-B, and JA, and activates the promoters of diterpenoid biosynthetic genes through the N box sequence. To explore whether *OsbHLH5* also has a role in regulating the biosynthesis of diterpenoid phytoalexins, this study detected the content of diterpenoid phytoalexins and the expression levels of their synthesis-related genes in both the overexpression materials and the CRISPR materials of *OsbHLH5*. The results confirm that *OsbHLH5* positively regulates the biosynthetic genes of diterpenoid phytoalexins (*OsCPS4*, *OsKSL4/7/10/11*), promoting the accumulation of momilactones in rice. Therefore, our results not only imply a functional divergence between *OsbHLH5* and *OsbHLH25* in regulating rice phytoalexins, but also enrich the understanding of the regulatory network of the bHLH gene family.

The specific evolutionary mechanisms underlying the functional divergence of *OsbHLH5* and *OsbHLH25* warrant further investigation. Additionally, it has been reported that, in plants, MYB, bHLH, and WD40 often form an MBW complex to synergistically regulate gene expression, such as in the phenylpropanoid metabolic branch pathway—anthocyanin synthesis genes [62]. It can be speculated that *OsbHLH5* may also require the assistance of other transcription factors, especially MYB family transcription factors, which have been reported to participate in the regulation of the synthesis of phenolamide metabolites during functional processes. Interestingly, in the promoter analysis, we found that abundant MYB recognition sites were distributed in the promoters of genes related to phenolamides and to diterpenoid phytoalexin synthesis (Figure 1C).

Rice (*Oryza sativa*) produces phenolamides and diterpenoids as major plant phytoalexins. Shen et al. identified two phenolamide metabolic gene clusters in rice that confer broad-spectrum disease resistance and demonstrated that phenolamide metabolites have a direct inhibitory effect on fungal growth [58,63]. Diterpenoid metabolites also exhibit inhibitory effects on a variety of pathogens, both by inhibiting the growth and spore germination of the rice blast fungus and by being secreted from rice roots into the soil, where they exhibit allelopathic effects by inhibiting the growth of surrounding plants [7,23]. This study found that *OsbHLH5* synergistically positively regulates the accumulation of phenolamide and diterpenoid phytoalexins in rice. Further pathogen inoculation experiments have shown that the overexpression of *OsbHLH5* significantly enhances rice resistance to *Xoo*, indicating that *OsbHLH5* may be a key gene involved in rice defense against pathogens and could be further utilized as a target for rice resistance breeding. Additionally, it has been reported that some members of the bHLH transcription factor are induced by JA [64]. *OsbHLH148* is involved in the regulation of JA gene expression for the initial response to drought tolerance in rice, interacting with the OsJAZ proteins to form the rice OsbHLH148-OsJAZ-OsCOI1 signaling module [65]. In this study, through induced expression profiling, we found that *OsbHLH5* is transcriptionally induced by JA in rice and is not induced by various other abiotic stresses, indicating that the JA signaling pathway, as an upstream signal, regulates *OsbHLH5*, which, in turn, regulates secondary metabolic pathways. It can be seen that the bHLH family transcription factors and JA signaling pathway genes form a complex and intertwined regulatory network, where they can act as both upstream regulatory signals and downstream response factors. In summary, our study provides evidence that *OsbHLH5* synergistically regulates the accumulation of phenolamides and diterpenoid phytoalexins in rice, participating in rice disease resistance processes (Figure 7).

## 4. Materials and Methods

### 4.1. Co-Expression Analysis and Promoter Cis-Acting Regulatory Element Analysis

For the co-expression analysis, we used the rice gene co-expression database RiceFREND (http://ricefrend.dna.affrc.go.jp/ (accessed on 8 November 2024)) to investigate genes co-expressed with phenolamide and DP biosynthetic genes [66].

We downloaded the 2 kb sequences upstream of the phenolamide and DP biosynthetic genes from the Rice Genome Annotation Project database (https://rice.uga.edu/ (accessed on 8 November 2024)) and analyzed their promoter regions using PlantCARE (http://bioinformatics.psb.ugent.be/webtools/plantcare/html/ (accessed on 8 November 2024)) to detect cis-acting regulatory elements.

### 4.2. Plant Growth and Treatments

The rice variety used in this study was Zhonghua 11 (ZH11), an important japonica rice variety with excellent genetic background, playing a significant role in rice genetics research and new variety breeding. Rice plants were grown in a growth chamber under long-day (LD) conditions (16 h light with a white light intensity of 120 µmol m^−2^s^−1^ at 30 °C/8 h dark at 25 °C), or in paddy field conditions with natural sunlight for the pathogen inoculation assay.

To analyze the expression pattern of OsbHLH5 in response to abiotic stress or phytohormones, two-week-old ZH11 seedlings were grown on half-strength MS liquid medium under LD conditions in a growth chamber, and then treated with ABA (spraying 100 µmol/L ABA on the leaves) [67], JA (spraying 100 µmol/L MeJA on the leaves) [67], IAA (spraying 100 µmol/L IAA on the leaves) [68], BR (spraying 10 µmol/L BR on the leaves) [68], cold (4 °C growth chamber) [67], salt (irrigation with 200 mmol/L NaCl solution) [67], and drought (irrigation with 20% PEG4000 solution) [64]. For UV-B radiation, the seedlings were transferred into a UV-B-radiating chamber (Philips, Netherlands, TL8W/302 nm narrow-band UV-B tube, fluence 11.06 KJ m^−2^d^−1^) without white light for the indicated times. The UV-B intensity was measured using a UV radiometer with the UV-295 detector from the photoelectric instrument factory of Beijing Normal University, China [69]. RNA samples were taken at 0, 1, 6, and 24 h for gene expression analysis. Triple biological replications were used in the RT–qPCR assay.

### 4.3. Plasmid Construction

The gateway method was used to construct the plasmid [70]. The full-length cDNA sequences of *OsbHLH5* or promoter sequences (~2 kb upstream of target genes) were amplified from rice (Nipponbare), and these primers were designed based on the sequence in the Rice Genome Annotation Project database (https://rice.uga.edu/ (accessed on 8 November 2024)) by Premier 5.0 (primer length of between 18 and 27 nucleotides, GC content of around 40–60%, melting temperature (Tm) in the range of 55–65 °C). The entry clone was obtained through BP recombination of the PCR product with pDONR207 (Invitrogen, Carlsbad, CA, USA). The error-free clones were obtained through sequencing analysis, and then subcloned into PJC34, PJF754, and PJG94 to obtain the overexpression vector, tobacco expression vector, and Luc reporter vectors, respectively, via LR recombination. The japonica rice variety ZH11 was used as a transformation recipient. The *OsbHLH5* mutants were obtained using the CRISPR-Cas9 method, as previously described [48]. The primer sequences used to generate the construct are listed in Table S3.

### 4.4. RNA Extraction and Expression Analyses

Reverse transcription–quantitative PCR (RT–qPCR) was performed using total RNA extracted with an RNA extraction kit (TRIzol reagent; Invitrogen, Carlsbad, CA, USA) according to the manufacturer’s instructions. Briefly, 5 µg of RNA was used to synthesize the first-strand cDNAs in 20 µL of the reaction mixture using the EasyScript One-Step gDNA Removal and cDNA Synthesis SuperMix (TransGen, Beijing, China). The quantification of the transcript abundance was performed using the SYBR Premix Ex Taq kit (TaKaRa, Tokyo, Japan) on the ABI 7500 real-time PCR system (Applied Biosystems, Foster City, CA, USA). The expression levels were normalized to the expression of the rice gene *OsUbc13* (*LOC_Os01g48280*). All of the RT–qPCR analyses were performed for three biological replicates. The primers for RT–qPCR are listed in Table S3.

### 4.5. Detection and Analysis of Metabolites

For metabolite profiling, leaves were collected from individual plants at the five-leaf stage and immediately frozen in liquid nitrogen. The freeze-dried samples were crushed using a mixer mill (MM 400; Retsch, Haan, Germany) with zirconia beads for 1 min at 30 Hz, and 0.1 g of the dry powder was extracted overnight at 4 °C with 1 mL of 70% aqueous methanol containing 0.1 mg/L lidocaine (internal standard). Following centrifugation at 10,000× *g* for 10 min, the lipid-soluble extracts were absorbed, and 0.4 mL of each extract was mixed and filtered (SCAA-104, 0.22 µm pore size; Angel, Shanghai, China) before the LC-MS analysis.

The content of phenolamides and diterpenoids was determined using scheduled multiple reaction monitoring (MRM) via an LC-ESI-QQQ-MS/MS system (LCMS-8060, SHIMADZU, Kyoto, Japan) as previously described [71]. The UPLC (Shim-pack UFLC SHIMADZU CBM30A system) conditions were as follows: column, shim-pack GISS C18 (pore size 1.9 µm, dimensions 2.1 × 100 mm); solvent system, water (0.04% acetic acid), acetonitrile (0.04% acetic acid); gradient program, 95:5 *v/v* at 0 min, 5:95 *v/v* at 12.0 min, 5:95 *v/v* at 13.2 min, 95:5 *v/v* at 13.3 min, 95:5 *v/v* at 15.0 min; flow rate, 0.40 mL min^−1^; temperature, 40 °C; injection volume: 2 µL. The ESI source operation parameters were as follows: nebulizing gas flow, 3 L min^−1^; heating gas flow, 10 L min^−1^; interface temperature, 500 °C; DL temperature, 250 °C; heat block temperature, 400 °C; drying gas flow, 10 L min^−1^. The recorded data were processed with LabSolutions 5.91 software. We optimized the precursor ions (Q1), product ions (Q3), and retention time of the aforementioned metabolites, specifically including target Q1 pre bias, target collision energy, and target Q3 pre bias. Detailed information is provided in Table S5. Unless otherwise noted, diterpenoids chemicals were purchased from Fisher Scientific (http://www.fisher.co.uk/ (accessed on 8 November 2024)); phenolamide chemicals were purchased from ChemFaces (https://www.chemfaces.cn/ (accessed on 8 November 2024)).

### 4.6. Phylogenetic Analysis

The homologous protein sequences of OsbHLH5 in Arabidopsis, rice, and maize were obtained from the NCBI (https://www.ncbi.nlm.nih.gov/ (accessed on 8 November 2024)) and aligned using the CLUSTALW (v.1.83) program with the default parameters [72] (Table S2). Phylogenetic trees were constructed using the neighbor-joining method based on the Poisson model (MEGA7, http://megasoftware.net/ (accessed on 8 November 2024)). Bootstrap values were estimated (with 1000 replicates) to assess the relative support for each branch. Gap treatment was undertaken with complete deletion.

### 4.7. Subcellular Localization

In the growth chamber, twelve-day-old seedlings (Nipponbare) grown on half-strength MS phytoagar medium were used for this experiment. The *OsbHLH5* coding sequence (without the stop codon) was cloned into the pM999-GFP vector to generate pM999-GFP-OsbHLH5 (p35S::*OsbHLH5*::*GFP*). The fusion construct p35S::*OsbHLH5*::*GFP* was co-transformed with p35S::GHD7::RFP, which was used as a nuclear marker, into rice protoplasts via polyethylene-glycol-mediated transformation, as previously described [67]. Briefly, the protoplasts were isolated in a digestion solution including 10 mM MES (pH 5.7), 0.6 M mannitol, 1 mM CaCl_2_, 3 mM bate-mercaptoethanol, 0.1% BSA, 0.75% Cellulase R10 (Yakult Pharmaceutical), and 0.75% macerozyme R10 for 5 h under dark conditions. The following collection and incubation were performed in W5 solution (2 mM MES, pH 5.7, 154 mM NaCl, 5 mM KCl, and 125 mM CaCl_2_) at room temperature, and then the protoplasts were filtered through a sieve mesh and resuspended in 4 mM MES, 0.6 mannitol, and 15 mM MgCl_2_ after centrifugation at 100 g for 5 min. Each transformation contained 10 mL of different plasmid, 100 mL of protoplasts, and 110 mL of polyethylene glycol–CaCl_2_ solution (0.6 M mannitol, 100 mM CaCl_2_, and 40% polyethylene glycol 4000). After incubation at room temperature for 10 min, 440 mL of W5 solution was added to stop the process. Protoplasts were cultured in 800 mL of 4 mM MES, pH 5.7, 0.6 mannitol, and 4 mM KCl after collection at 100 g for 5 min on 24-well culture plates. After 12 h of incubation at 22 °C, fluorescence was observed under a confocal microscope (Olympus FV1200, Tokyo, Japan).

### 4.8. Pathogen Inoculation Assays

To evaluate bacterial blight disease, rice plants (including wild-type ZH11, *OsbHLH5*-OX, and *Osbhlh5*-CR lines), grown in paddy field conditions with natural sunlight until the 5-leaf stage, were inoculated with PXO99 using the leaf-clipping method. *Xoo* strains of PXO99 were grown on nutrient agar medium (0.1% yeast extract, 0.3% beef extract, 0.5% polypeptone, and 1% sucrose) at 28 °C for 2 days. The samples were resuspended and diluted with sterile water to *A*_600nm_ = 0.5 for inoculation. Five to ten inoculated leaves were scored by measuring the lesion length at approximately 14 days post-inoculation (dpi). The inoculated leaves were photographed using a scientific scanner (Image Scanner III, GE, Gothenburg, Sweden).

### 4.9. Dual-Luciferase Transcriptional Activity Assays

Promoters of candidate target genes (~2 kb upstream of these genes) were amplified from Nipponbare (a type of conventional japonica rice variety known for its widespread use in scientific research on rice) and cloned into the modified pH2GW7 vector (PJG094) containing the firefly luciferase (fLUC) gene and the Renilla luciferase gene (rLUC) as reporters, while the full-length cDNA of *OsbHLH5* was cloned into the PJF754 (pEAQ-HT-DEST2) vector as an effector. The plasmids were transferred into *A. tumefaciens* EHA105 via electroporation and co-infiltrated into four-week-old *N. benthamiana* leaves. The luciferase activities were measured using the Dual-Luciferase Reporter Assay System (Promega, Madison, WI, USA) according to the manufacturer’s instructions, and the LUC activity was normalized to the REN activity.

### 4.10. Statistical Analysis

The data were analyzed using Microsoft Office Excel 2010 and SPSS 23.0 (SPSS, IBM, Chicago, IL, USA). The results are expressed as means ± SD of at least three independent experiments. The differences among groups were determined using a two-tailed Student’s *t* test.

## 5. Conclusions

Phenolamide and diterpenoid phytoalexins are important classes of plant defense compounds. However, the regulatory factors that coordinate their biosynthesis remain unclear. Here, through co-expression analysis and phylogenetic tree analysis, we identified *OsbHLH5* as a factor that co-regulates the accumulation of phenolamide and diterpenoid phytoalexins in rice, contributing to disease resistance. Our findings provide new insights into the metabolic interactions during pathogen response and offer novel genetic resources for the development of new rice varieties with enhanced resistance through genetic improvement.

## Data Availability

All data are available upon reasonable request.

## Supplementary Materials

The following supporting information can be downloaded at https://www.mdpi.com/article/10.3390/ijms252212152/s1.
