# Supplementary material for: OsbHLH5 Synergically Regulates Phenolamide and Diterpenoid Phytoalexins Involved in the Defense of Rice Against Pathogens
Source: Int J Mol Sci. 2024 Nov 12;25(22):12152. doi: 10.3390/ijms252212152 (PMC11595221; doi:10.3390/ijms252212152)
Supplement: Supplementary file 1 [file ijms-25-12152-s001.zip › ijms-3240562 supplementary.pdf]

Supplemental Figures and Tables

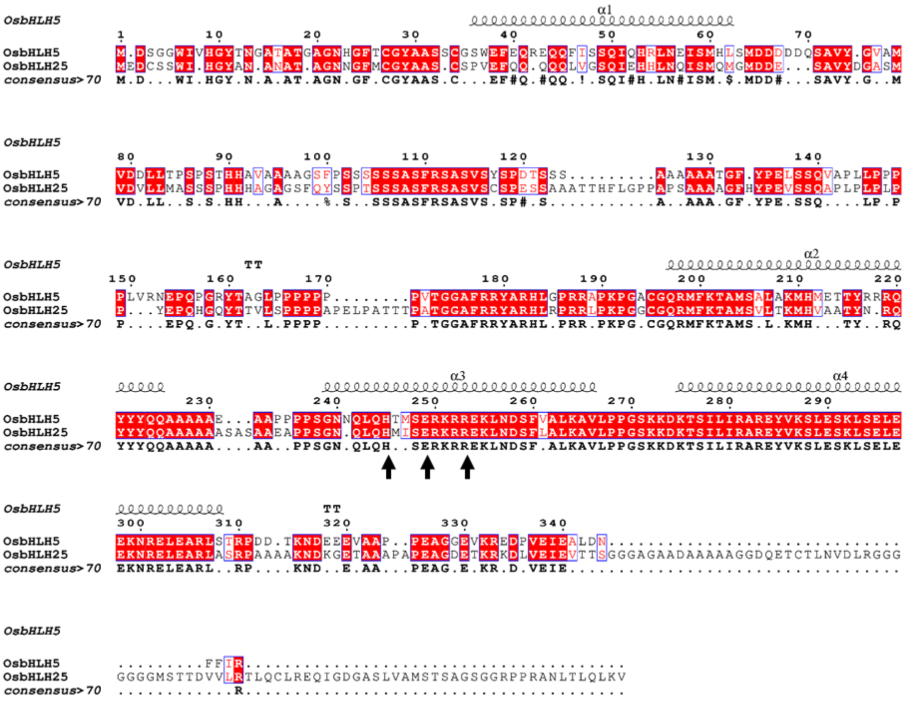

Figure S1. Amino acid sequence comparison of *OsbHLH5* and *OsbHLH25*.

The black arrow marks the typical triad of residues, His, Glu, and Arg, located in the basic regions.

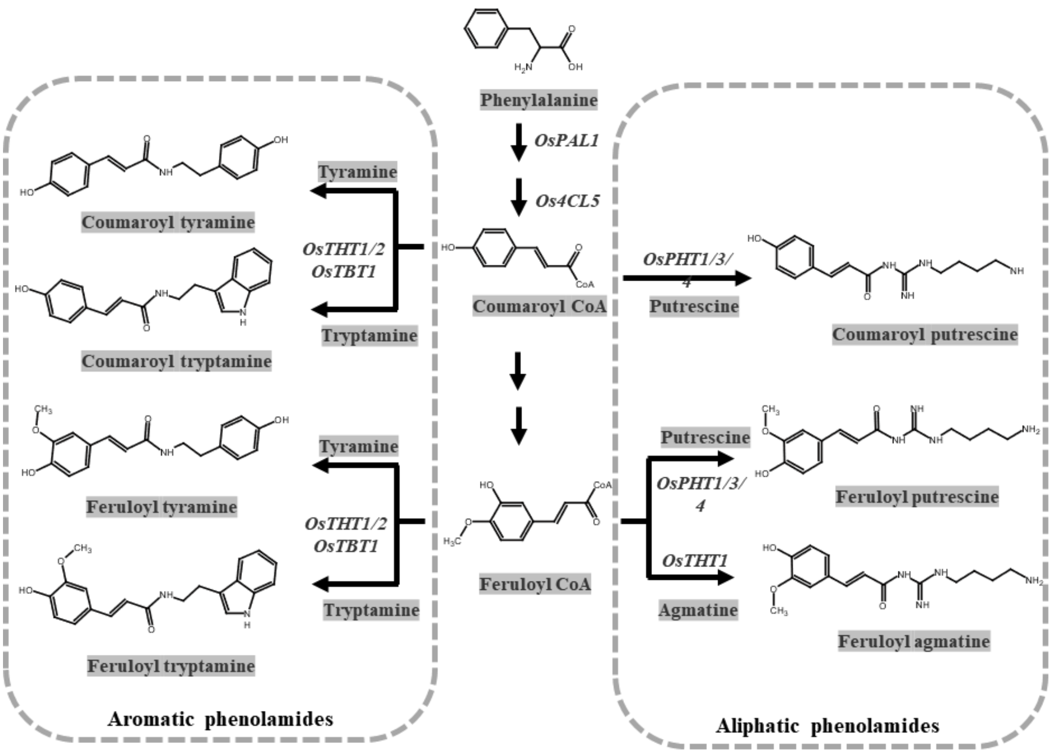

Figure S2. Biosynthetic pathways of phenolamides in rice.

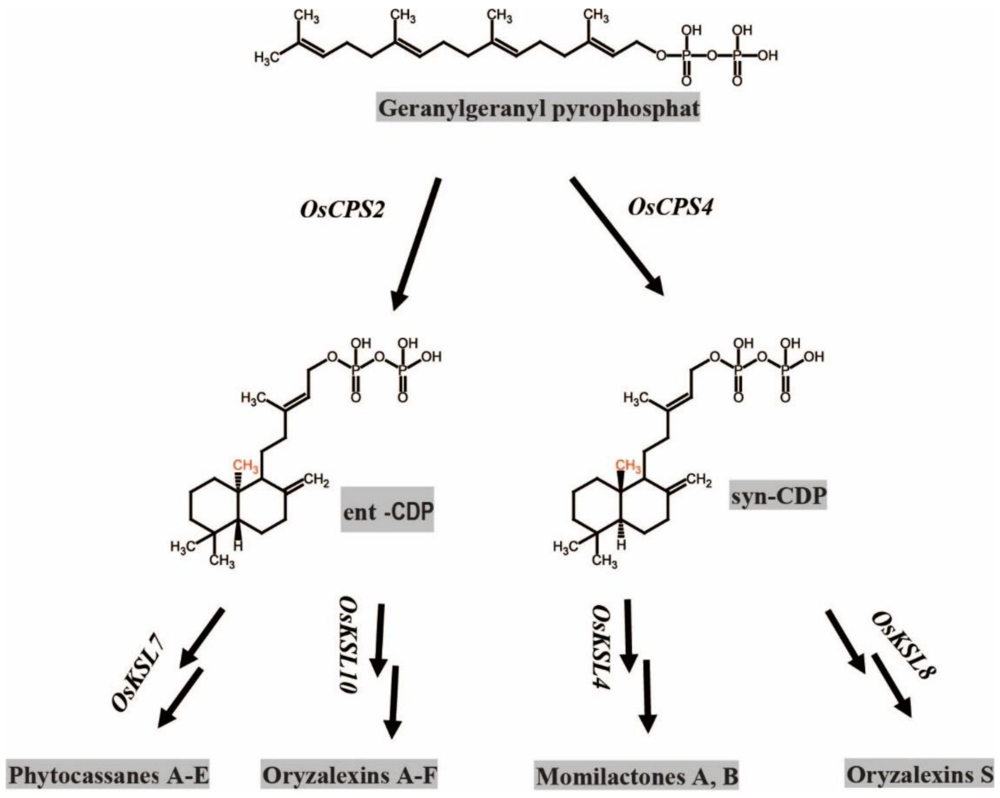

Figure S3. Biosynthetic pathways of diterpenoids in rice.
